# CRISPR/Cas9-generated p47^*phox*^-deficient cell line for Chronic Granulomatous Disease gene therapy vector development

**DOI:** 10.1038/srep44187

**Published:** 2017-03-13

**Authors:** Dominik Wrona, Ulrich Siler, Janine Reichenbach

**Affiliations:** 1 Division of Immunology, University Children’s Hospital Zurich, Zurich, Switzerland; 2 Children’s Research Center, Zurich, Switzerland; 3 University of Zurich, Zurich, Switzerland

## Abstract

Development of gene therapy vectors requires cellular models reflecting the genetic background of a disease thus allowing for robust preclinical vector testing. For human p47^phox^-deficient chronic granulomatous disease (CGD) vector testing we generated a cellular model using clustered regularly interspaced short palindromic repeats (CRISPR)/Cas9 to introduce a GT-dinucleotide deletion (ΔGT) mutation in p47^phox^ encoding *NCF1* gene in the human acute myeloid leukemia PLB-985 cell line. CGD is a group of hereditary immunodeficiencies characterized by impaired respiratory burst activity in phagocytes due to a defective phagocytic nicotinamide adenine dinucleotide phosphate (NADPH) oxidase. In Western countries autosomal-recessive p47^phox^-subunit deficiency represents the second largest CGD patient cohort with unique genetics, as the vast majority of p47^phox^ CGD patients carries ΔGT deletion in exon two of the *NCF1* gene. The established PLB-985 *NCF1* ΔGT cell line reflects the most frequent form of p47^phox^-deficient CGD genetically and functionally. It can be differentiated to granulocytes efficiently, what creates an attractive alternative to currently used iPSC models for rapid testing of novel gene therapy approaches.

Chronic Granulomatous Disease (CGD) comprises a group of monogenetic immunodeficiencies characterized by impaired respiratory burst activity and microbicidal activity of phagocytes leading to recurrent life-threatening infections^1^. CGD can be cured by hematopoietic stem cell transplantation (HSCT)^2^. Alternatively, autologous retroviral gene therapy has been used in selected patients with X-linked CGD (X-CGD)^3^,^4^,^5^,^6^,^7^.

Mutations in gp91^phox^, p47^phox^, p67^phox^, p40^phox^ or p22^phox^ subunits of the phagocytic nicotinamide adenine dinucleotide phosphate (NADPH) oxidase may cause CGD^8^. In Western countries gp91^phox^-deficiency is the most frequent from (65%)^9^, followed by p47^phox^-deficiency (25%). Mutations within gp91^phox^ are scattered throughout the whole cytochrome b-245 beta chain (*CYBB*) gene. Conversely, p47^phox^-deficiency is almost exclusively caused by a single GT-dinucleotide deletion (ΔGT) in exon 2 of neutrophil cytosolic factor 1 (*NCF1*) gene causing frameshift and premature translation stop. The ΔGT deletion is shared with two pseudogenes, *NCF1B* and *NCF1C*, all located on the same chromosome sharing extraordinary homology (99.5%) (Fig. 1A,B). Presumably, homologous recombination causes the predominance of the ΔGT mutation in p47^phox^-deficient CGD patients^10^.

Currently, development of gene therapy vectors for p47^phox^-deficient CGD is hampered by the absence of human cell lines for rapid gene therapy vector testing. p47^phox^−/− mouse models exist, but cannot replace vector testing on human cells. Recently, we established human p47^phox^-deficient induced pluripotent stem cell (iPSC) lines harboring the ΔGT within the *NCF1* gene^11^. Only these iPSC-based cell lines reflect the genetic background of the most frequent mutation in CGD. As maintenance and differentiation of iPSC lines is laborious and in many aspects impractical, we established a novel model for ΔGT p47^phox^-deficient CGD based on a human acute myeloid leukemia PLB-985 cell line^12^.

We nucleofected PLB-985 wild type (WT) cells with pPX458-*NCF1* plasmid encoding Cas9, single guide RNA (sgRNA) targeting *NCF1*, and GFP. To introduce the ΔGT in *NCF1* a 100-nucleotide long single stranded oligonucleotide (ssODN) template, carrying the ΔGT, was co-transfected (Fig. 1C).

Nucleofected GFP expressing cells were sorted by fluorescence-activated cell sorting (FACS) and expanded to monoclonal cell lines. Out of 609 sorted cells 22 clones could be expanded (3.6% survival rate, Supplementary Table S1). These clones were analyzed by PCR co-amplification of *NCF1, NCF1B* and *NCF1C* alleles (Fwd1, Rev1 primers, Fig. 1B), followed by BsrG1 digestion^13^. BsrG1 cleavage of *NCF1* gene gives 135bp and 63bp products, while *NCF1B, NCF1C*, or mutated *NCF1* lack the BsrG1 restriction site (Fig. 1C).

The BsrG1 analysis of one nucleofected PLB-985 clone displayed the same band pattern as of a ΔGT p47^phox^-deficient CGD patient (Fig. 1D and Supplementary Fig. S1), suggesting a Cas9-mediated disruption of the BsrG1 site in both *NCF1* alleles (homozygous mutation efficiency 4.5%). The BsrG1 digestion analysis of the remaining clones suggested heterozygosity in these clones (Supplementary Fig. S1).

To confirm the presence of the ΔGT in mutated NCF1, genomic DNA of WT and pPX458-*NCF1*-treated PLB-985 cells were used for PCR co-amplification of the *NCF1, NCF1B*, and *NCF1C* (Fwd1, Rev2 primers, Fig. 1B). The barcoded PCR products were analyzed by single molecule real-time sequencing (SMRT-seq) for the presence of GT-dinucleotide, ΔGT, as well as for the presence of one copy of 20-nucleotide (20-nt) intronic repeat (1 × 20nt) derived from *NCF1* or two repeats (2 × 20nt) derived from *NCF1B* or *NCF1C* (Fig. 1B). SMRT-seq analysis showed that PLB-985 WT and PLB-985 *NCF1* ΔGT cell lines displayed almost identical percentage (61%) of reads with one or two copies of the 20-nucleotide repeat (Fig. 1E), indicating that all three *NCF1* loci were co-amplified with comparable efficiencies. Moreover, all SMRT-seq reads from PLB-985 *NCF1* ΔGT displayed the ΔGT, while in PLB-985 WT 37.4% harbored the GT-dinucleotide sequence, as expected from the two *NCF1* alleles.

Potential off-target sites of utilized sgRNA were predicted (Supplementary Table S2) and DNA sequences of 14 sites with highest scores were analyzed for presence of insertions or deletions (indels) by Surveyor assay. In none of the potential off-target sites indels were observed (Supplemetary Fig. S2).

Flow cytometry analysis of granulocytically differentiated PLB-985 WT and PLB-985 X-CGD cells^14^ revealed 86.1% and 89.4% of p47^phox^ protein expressing cells (Fig. 2A) (gating, Supplementary Fig. S3). No p47^phox^ expression was observed in differentiated PLB-985 *NCF1* ΔGT cells, which were CD14-negative, CD15-positive and partially gp91*^phox^*-positive (Supplementary Fig. S4).

To confirm that differentiated PLB-985 *NCF1* ΔGT cells mirror the absent respiratory burst observed in primary neutrophils of ΔGT p47^phox^-deficient CGD patients, we tested this cell line for NADPH oxidase-mediated superoxide production with nitroblue tetrazolium (NBT) test. In differentiated PLB-985 WT cells, phorbol-12-myristate-13-acetate (PMA)-stimulation induced superoxide production in 47.8 ± 4.2% of cells, as was visualized by formazan precipitates (Fig. 2B and Supplementary Fig. S5). In contrast, PMA-stimulation of differentiated PLB-985 X-CGD and PLB-985 *NCF1* ΔGT cells resulted only in background signal of 1.7 ± 1.0% and 0.9 ± 0.5%. These results indicate that the established p47^phox^-deficient PLB-985 *NCF1* ΔGT cell line does not respond to PMA-stimulation by reactive oxygen species (ROS) production. The absence of ROS production in PLB-985 X-CGD and PLB-985 *NCF1* ΔGT cells was confirmed by chemiluminescence assay (Fig. 2C).

The iPSC model of ΔGT p47^phox^-deficient CGD^11^ can be differentiated to monocytes, macrophages^11^ and granulocytes^15^,^16^. Nevertheless, the use of iPSCs is laborious, and time-consuming, as phagocytic differentiation requires long culture periods of 35–43 days^15^,^17^ requiring large amounts of cytokines and continuous surveillance of the culture. In contrast full granulocytic differentiation of PLB-985 *NCF1* ΔGT cell line takes 7 days, requires only fetal calf serum (FCS) restriction (5%) and supplementation with 0.5% N,N-dimethylformamide (DMF). Furthermore, the differentiation of neutrophils can be easily assessed by flow cytometry (Supplementary Fig. S3).

The function of the NADPH oxidase complex within PLB-985 *NCF1* ΔGT cells was reconstituted by transduction with two γ-retroviral vectors encoding p47^phox^ protein (Fig. 2D). Transgene expression was driven either by the ubiquitously active spleen focus-forming virus (SFFV) promoter or by the myelospecific microRNA 223 (mir223) promoter^18^. The expression cassette was composed of truncated Low-affinity nerve growth factor receptor (ΔLNGFR) and p47^phox^ linked by the 2 A self-cleaving peptide^19^. Transduction efficiency reached 8.9% and 11.6% for mir223, and SFFV-driven γ-retroviral vectors (Fig. 2E).

Functional reconstruction of the NADPH oxidase complex activity in transduced and differentiated PLB-985 *NCF1* ΔGT cells was assessed by NBT assay (Fig. 2F) and chemiluminescence assay (Fig. 2G). Percentage of NBT-positive cells reached 13.4 ± 2.1% with the mir223, and 11.8 ± 1.8% with the SFFV-driven ΔLNGFR-2A-p47^phox^ expression constructs (Fig. 2F and Supplementary Fig. S5). Chemiluminescence assay of transduced and differentiated PLB-985 *NCF1* ΔGT cells confirmed reconstitution of ROS production. These results clearly indicate that the defect of the NADPH oxidase complex caused by the ΔGT mutation within *NCF1* in the PLB-985 *NCF1* ΔGT cell line can be corrected.

In summary, the CRISPR/Cas9-generated PLB-985 *NCF1* ΔGT cell line fully recapitulates the genetic background and functional NADPH oxidase defect found in majority of p47^phox^-deficient CGD patients, can be corrected genetically and differentiated into functional neutrophils. The PLB-985 *NCF1* ΔGT cell line therefore represents a promising cost-effective tool for rapid gene therapy vector testing.

## Materials and Methods

### Reagents and antibodies

Single stranded DNA was purchased from Microsynth (Balgach, Switzerland). RPMI 1640 medium with stable glutamine and fetal calf serum (FCS) were purchased from PAN-Biotech (Aidenbach, Germany). Penicillin/streptomycin, Phusion High-Fidelity DNA Polymerase, and dNTPs were obtained from Thermo Fisher (Reinach, Switzerland), SCR7 was from Biovision (Milpitas, CA). BsrG1 was derived from New England Biolabs (Frankfurt/Main, Germany). N,N-dimethylformamide (DMF), propidium iodide (PI) and nitro blue tetrazolium (NBT) were obtained from Sigma Aldrich (Buchs, Switzerland). Mouse anti-human ΔLNGFR monoclonal antibody, clone ME20.4–1.H4, fluorescein isothiocyanate (FITC)-conjugated was purchased from Miltenyi Biotec (Bergisch Gladbach, Germany), and mouse anti-human p47^phox^ monoclonal antibody clone 1 was allophycocyanin (APC)-conjugated by Becton Dickinson AG (Allschwil, Switzerland). PECy7-conjugated anti-mouse/human CD11b antibody was from BioLegend (Fell, Germany). Mouse anti-human CD15 FITC (Catalog no 347423) and mouse anti-human CD14 FITC (Catalog no. 345784) were from Beckton Dickinson AG (Allschwil, Switzerland). Mouse anti-Flavocytochrome b558 (human)-FITC clone 7D5 recognizing gp91^phox^ was from LabForce AG (Muttenz, Switzerland).

### Plasmid preparation

The sgRNA sequence targeting the *NCF1* gene (but not *NCF1B* and *NCF1C* pseudogenes) was designed using Optimized CRISPR Design (http://crispr.mit.edu, F. Zhang laboratory, MIT 2015). Guide sequence CCCCCAG**GTGT**ACATGTTCC was cloned into pSpCas9(BB)-2A-GFP (PX458) (F. Zhang, Addgene plasmid #48138)^20^ to generate pPX458-*NCF1*, which was confirmed by sequencing (Microsynth).

### Cell culture and differentiation

We utilized PLB-985 cell line^12^, a subclone of HL-60^21^,^22^, as it is capable of granulocytic differentiation. Cells were cultured in RPMI 1640 medium supplemented with 10% (vol/vol) FCS, and 1% (vol/vol) penicillin/streptomycin in a humidified incubator at 37 °C and 5% CO_2_. Granulocytic differentiation of logarithmically growing PLB-985 cells at density of 0.8·10^6^ cells/mL was induced by reduction of the FCS content to 5%, and supplementation with 0.5% (vol/vol) DMF. After 3 days, an equivalent of initial volume of differentiating medium was added and the differentiation continued until day 7.

### CRISPR/Cas9 mediated generation of PLB-985 *NCF1* ΔGT cell line

2·10^6^ PLB-985 wild type (WT) cells were nucleofected (Amaxa Cell Nucleofector Kit V and Amaxa Nucleofector II, program C-023 (Lonza, Basel, Switzerland)) with 40 μg of the pPX458-*NCF1* and a 100-nucleotide long ssODN sequence at final concentration 3 μM. The sequence of the ssODN was: GCC TCT TTG GAG GCT GAA TGG GGT CCC CCG ACT CTG GCT TTC CCC CAG **GT**A CAT GTT CCT GGT GAA ATG GCA GGA CCT GTC GGA GAA GGT GGT CTA CCG G. Immediately after nucleofection 500 μL of medium was added to the cuvette and the cells were incubated at room temperature for 10 minutes, transferred to 10 mL medium, then cultured for 48 hours. Additionally, the culture was supplemented with 1 μM SCR7 starting 3–4 hours post nucleofection. After 48 hours GFP expressing cells were sorted with FACS Aria III FCF (Becton Dickinson AG, Allschwil, Switzerland) into single wells with 100 μL of pre-conditioned, sterile filtered medium supplemented with 1 μM SCR7.

### DNA Amplification and Restriction Fragment Length Polymorphism Analysis

**Figure 1:**
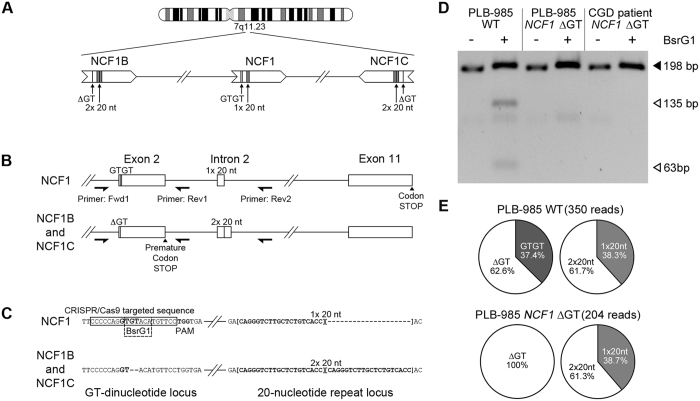
NCF1 loci in PLB-985 cell lines. (**A**) *NCF1* gene is flanked by two pseudogenes, *NCF1B* and *NCF1C*. (**B**) Differentiators between *NCF1* and pseudogenes. PCR primers: Fwd1, Rev1, Rev2. (**C**) DNA sequence of GT-dinucleotide and 20 nucleotide repeat loci. CRISPR/Cas9-targeted sequence, protospacer adjacent motif (PAM) and BsrG1 site: rectangles; 20-nucleotide repeat: squared brackets. (**D**) Loss of BsrG1 restriction site in PCR amplified *NCF1* (**B**,**C**) exon 2 (primers Fwd1 and Rev1 (**B**)) in PLB-985 *NCF1* ΔGT cells. Full-length gel is presented in Supplementary Fig. S6. (**E**) Percentages of reads from SMRT-seq of PCR products (primers Fwd1 and Rev2 (**B**)). The absolute number of analyzed SMRT sequencing reads which have passed all filtering criteria is given.

Processing of human samples is covered by ethical vote KEK ZH nr. 2015/0135. Genomic DNA was isolated using DNeasy Blood & Tissue Kit (Qiagen, Hombrechtikon, Switzerland). Parallel PCR co-amplification of the *NCF1, NCF1B*, and *NCF1C* was performed using published primers^23^ (Fwd1 and Rev1, Fig. 1B). For PCR reaction using Fwd1/Rev2 primers Fwd1 primer was barcoded for each template producing products of 417 bp from *NCF1* and of 435 bp from *NCF1B* and *NCF1C*. PCR products were pooled for SMRT sequencing and analyzed according to barcode identities.

The PCR mixture included HF 10× buffer, 200 μM of each dNTP, 240 nM of each primer, 0.04 U/μL of Phusion High-Fidelity DNA Polymerase, and 2.5 ng/μL of genomic DNA. Initial denaturation at 95 °C for 3 minutes was followed by 36 cycles of denaturation (95 °C, 30 seconds), annealing (70 °C, 30 seconds), elongation (72 °C, 8 seconds), and a final elongation step (72 °C, 1 minute). PCR products were digested with BsrG1 and visualized on a 3% (w/vol) agarose gel.

### Single molecule real-time (SMRT) sequencing

The *NCF1* gene and *NCF1B* and *NCF1C* pseudogenes were PCR amplified in parallel utilizing primers Fwd1 and Rev2 (Fig. 1B)^23^. The primer Fwd1 was barcoded for each FACS sorted clone and obtained PCR products were of 417 bp for *NCF1* and 435 bp for *NCF1B* and *NCF1C* templates. PCR reaction consisted of initial denaturation (95 °C, 3 minutes), 40 cycles of denaturation (95 °C, 30 seconds), annealing (60 °C, 30 seconds), elongation (72 °C, 30 seconds), and a final elongation step (72 °C, 3 minutes). PCR products were gel purified using QIAquick Gel Purification Kit (Qiagen). 10–20 ng of gel-purified PCR products were pooled and analyzed by SMRT sequencing by Functional Genomics Center Zurich, ETH/University of Zurich, Zurich, Switzerland. Briefly, DNA amplicon librabry was produced using DNA Template Prep Kit 1.0 (Pacific Biosciences, Menlo Park, California, United States). The input DNA concentration and quality was measuered using Qubit Fluorometer dsDNA Broad Range assay (Life Technologies, Zug, Switzerland) and Bioanalyzer 2100 12 K DNA Chip assay (Agilent Technologies AG, Basel, Switzerland). The SMRT bell template was prepared by end-repair of the DNA amplicons, followed by blunt-end ligation of overhang adapters and exonuclease treatment. The SMRT bell template was complexed with polymerase using P6 DNA/Polymerase Binding Kit 2.0 (Pacific Biosciences) according to the manufacturer’s instructions. The samples were sequenced using Pacific Biosciences RS2 platform. From the raw reads, high quality circular consensus reads (CCS) were obtained through the Reads Of Insert protocol available in the SMRT Analysis suite (Pacific Biosystems). CCS reads were then de-multiplexed by exact matching of the sample barcodes, starting at the base immediately preceeding the Fwd1 primer sequence. Reads with a length between 400 and 450 nucleotides were retained and matched against the *NCF1* reference sequence using blast^24^.

### Surveyor Assay

Off-target sites were predicted utilizing the Optimized CRISPR Design (http://crispr.mit.edu, F. Zhang laboratory, MIT 2015). Loci of predicted sites (see Supplementary Table S2) were PCR amplified from gDNA of PLB-985 WT and PLB-985 *NCF1* ΔGT cells. Corresponding PCR amplification products were mixed in a ratio of 1:1, while PCR amplification product of PLB-985 WT was used as a control. The samples were denatured at 95 °C for 10 minutes, slowly renatured, and digested using Surveyor^®^ Mutation Detection Kit For Standard Gel Electrophoresis (Integrated DNA Technologies, Leuven, Belgium) according to the manufacturer’s instructions.

### Data Availability

All SMRT sequencing data can be obtained upon request.

### Virus production and transduction

Viruses were produced as described previously^19^. In brief, 293T cells were co-transfected with pUMVC, pMD2.VSV.G and γSIN-SFFV-ΔLNGFR-2A-p47^phox^ 19 or γSIN-miR223-ΔLNGFR-2A-p47^phox^ 18 in presence of 10 mM chloroquine. Sterile filtered virus containing supernatants were concentrated using Amicon-15 centrifugal filter devices with 100 kDa cutoff and stored at −80 °C until use.

PLB-985 *NCF1* ΔGT cells were γ-retrovirally transduced with vectors co-encoding p47^phox^ protein and low-affinity nerve growth factor receptor (ΔLNGFR)^18^,^19^ at concentration of 0.8·10^6^ cells/mL, at multiplicity of infection (MOI) = 5, and in presence of 8 μg/mL protamine sulfate by spinoculation at 1286 g, 32 °C for 90 minutes, followed by incubation at 37 °C and 5% CO_2_ for 24 hours. Then, cells were differentiated into granulocytes in presence of 0.5% DMF and 5% FCS for 6 days and analyzed thereafter.

### Flow cytometry analysis

ΔLNGFR surface staining was carried out with 6 μL of mouse anti-LNGFR-FITC (Miltenyi Biotec) per 5 * 10^5^ cells in 100 μL for 20 minutes, followed by washing in PBS. Intracellular staining of p47^phox^ was conducted utilizing 360 ng of mouse anti-p47^phox^-APC (clone 1) per 5 * 10^5^ cells in 100 μL and the IntraCell Kit (Immunostep, Salamanca, Spain) according to the manufacturer’s instructions. Flow cytometry analysis was performed using a Gallios Flow Cytometer (Beckman Coulter).

### Nitroblue-tetrazolium (NBT) test

Differentiated PLB-985 WT, PLB-985 X-CGD, and PLB-985 *NCF1* ΔGT cells, as well as transduced PLB-985 *NCF1* ΔGT cells were incubated in growth medium supplemented with 100 μg/mL NBT in the presence of 200 ng/mL PMA at 37 °C and 5% CO_2_ for 30 minutes. Subsequently, the cells were fixed in 1% (vol/vol) formaldehyde. 250 cells per cytospin slide were analyzed manually for NBT activity using a Leica DM IL Fluo light microscope equipped with a DFC420 digital camera and LEICA application suite acquisition software (Leica Microsystems AG, Glattbrugg, Switzerland).

### Chemiluminescence assay

Chemiluminescence assay on differentiated PLB-985 WT, PLB-985 X-CGD, and PLB-985 *NCF1* ΔGT cells, as well as transduced PLB-985 *NCF1* ΔGT was conducted in 96-well plate format at a cell density of 1 * 10^5^ cells/200 ml in a Mithras LB 940 Luminometer (Berthold Technologies GmbH, Zug, Switzerland) as described recently^11^.

## Additional Information

**How to cite this article**: Wrona, D. *et al*. CRISPR/Cas9-generated p47^*phox*^-deficient cell line for Chronic Granulomatous Disease gene therapy vector development. *Sci. Rep.* **7**, 44187; doi: 10.1038/srep44187 (2017).

**Publisher's note:** Springer Nature remains neutral with regard to jurisdictional claims in published maps and institutional affiliations.

## Supplementary Material

Supplementary Information

## Figures and Tables

**Figure 2:**
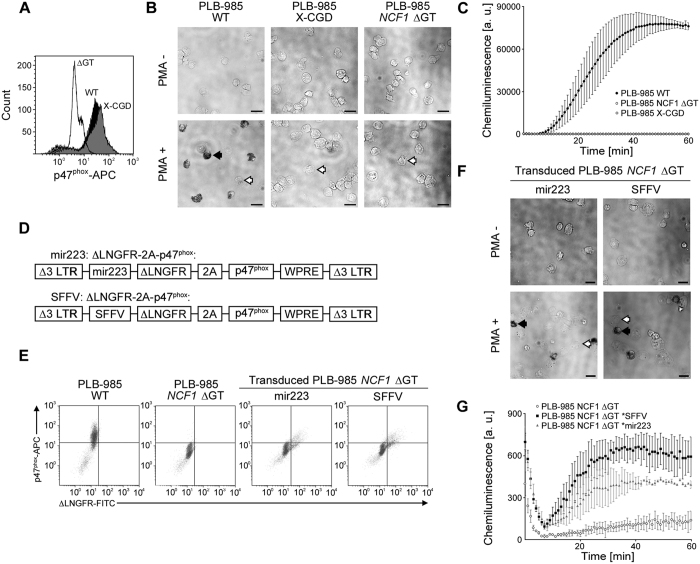
Superoxide production in PLB-985 *NCF1* ΔGT. (**A**) p47^phox^ flow cytometry analysis and(**B**) ROS production in granulocytically differentiated PLB-985, PLB-985 X-CGD and PLB-985 *NCF1* ΔGT cells measured by NBT test. NBT-positive: black arrow; NBT-negative: white arrows. Scale bar: 25 μm.(**C**) Chemiluminescence assay of un-transduced PLB-985 WT, PLB-985 *NCF1* ΔGT, and PLB-985 X-CGD cell line (mean and standard deviation of mean, n = 3, a.u. = arbitrary units). (**D**) γ-retroviral vectors for reconstitution of p47^phox^ expression. (**E**) Flow cytometry analysis of p47^phox^ and ΔLNGFR expression in differentiated un-transduced PLB-985 WT and PLB-985 *NCF1* ΔGT, and transduced PLB-985 *NCF1* ΔGT cells. (**F**) ROS production in transduced PLB-985 *NCF1* ΔGT cells upon differentiation, measured by NBT test. NBT-positive: black arrow; NBT-negative: white arrows. Scale bar: 25 μm. (**G**) Chemiluminescence assay of transduced PLB-985 *NCF1* ΔGT cells (mean and standard deviation of mean, n = 3).
