# Supplementary material for: CRISPR/Cas9-generated p47phox-deficient cell line for Chronic Granulomatous Disease gene therapy vector development
Source: Sci Rep. 2017 Mar 13;7:44187. doi: 10.1038/srep44187 (PMC5347011; doi:10.1038/srep44187)
Supplement: Supplementary Information [file srep44187-s1.pdf]

Supplementary Information

CRISPR/Cas9-generated p47phox-deficient cell line for

Chronic Granulomatous Disease gene therapy vector development

Authors

Dominik Wrona[1,2], Ulrich Siler[1,2*] and Janine Reichenbach[1,2*]

[1] Division of Immunology, University Children's Hospital Zurich, Zurich, Switzerland

[2] Children's Research Centre, Zurich, Switzerland

* Equal contribution

Supplementary Figures

Supplementary Figure S1

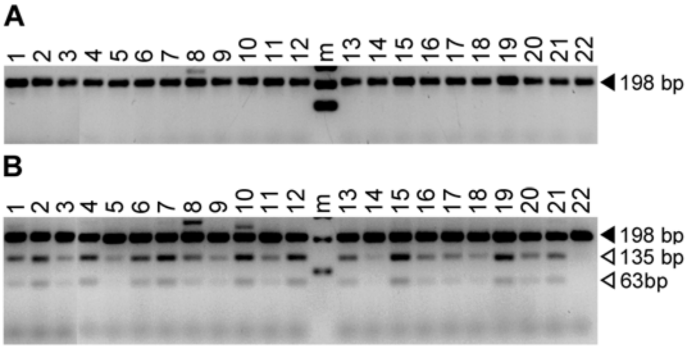

Restriction Fragment Length Polymorphism of single clones of CRISPR/Cas9-treated PLB-985.

PLB-985 cell line was transfected with CRISPR/Cas9 GFP co-expression plasmid and ssODN, followed by FACS sorting to monoclonality. The NCF1 gene and pseudogenes in individual clones were analyzed by restriction fragment length polymorphism. (A) Undigested PCR co-amplification products of NCF1 and its pseudogenes for all individual clones (B) Only digestion of PCR products derived from WT NCF1 sequence with BsrG1 restriction enzyme led to appearance of two bands of size 135 bp and 63 bp. Only clone 22 showed complete lack of these two bands suggesting that both alleles of NCF1 were mutated.

Supplementary Figure S2

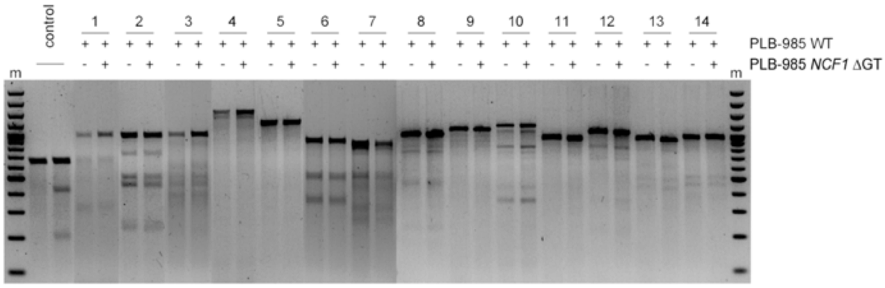

Surveyor Assay for detection of Cas9 introduced off-target mutations.

Surveyor Assay of 14 potential off-target sites of utilized CRISPR/Cas9 sgRNA. For each analyzed site no additional cleavage was observed, indicating no off-target activity of Cas9 in the PLB-985 *NCF1* ΔGT cell line.

Supplementary Figure S3

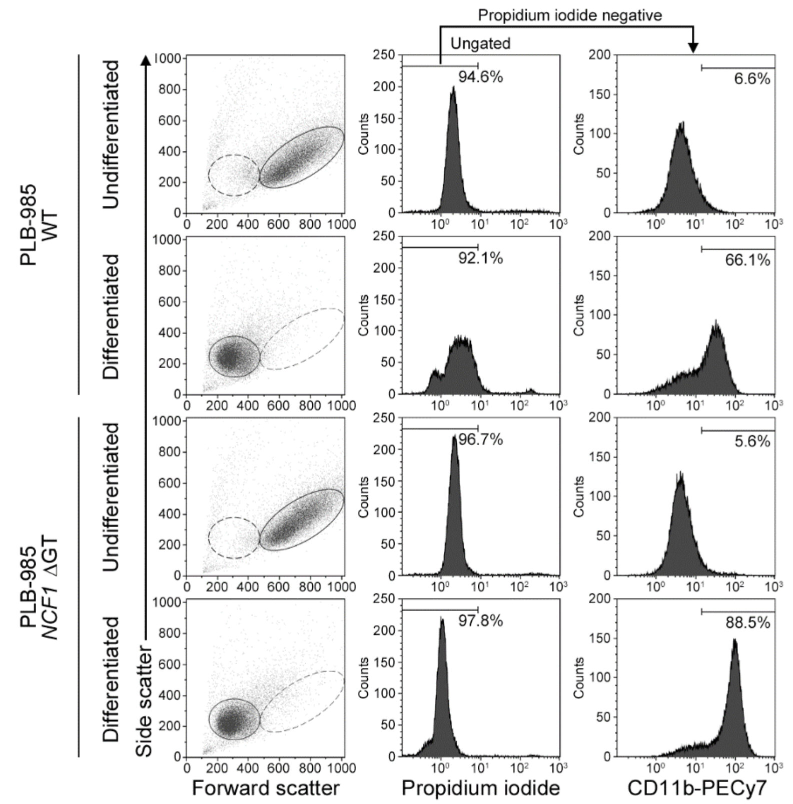

Flow cytometry analysis of differentiation and viability of PLB-985 WT and PLB-985 *NCF1* ΔGT cells.

WT PLB-985 and PLB-985 *NCF1* ΔGT cells were differentiated to granulocytes for 6 days followed by PMA stimulation for 5 minutes. Analysis of viability and differentiation status by propidium iodide (PI) staining (1 μg/ml) in ungated cells, and CD11b surface staining of PI-negative cells. DMF-induced granulocytic differentiation of PLB-985 cells is associated with a shift in forward/side scatter distribution. No difference has been observed in forward scatter versus side scatter plot between WT PLB-985 and PLB-985 *NCF1* ΔGT cells. PI staining showed that majority of analyzed cells were viable (PI-negative: 94.6 % PLB-985 WT undifferentiated, 92.1 % PLB-985 WT differentiated, 96.7 % PLB-985 *NCF1* ΔGT undifferentiated, and 97.8 % PLB-985 *NCF1* ΔGT differentiated), whilst CD11b presentation represents differentiation status

(undifferentiated PLB-985 WT and PLB-985 *NCF1* ΔGT: 6.5% and 5.6% CD11b-positive; differentiated PLB-985 WT and PLB-985 *NCF1* ΔGT: 66.1% and 88.5% CD11b-positive).

Supplementary Figure S4

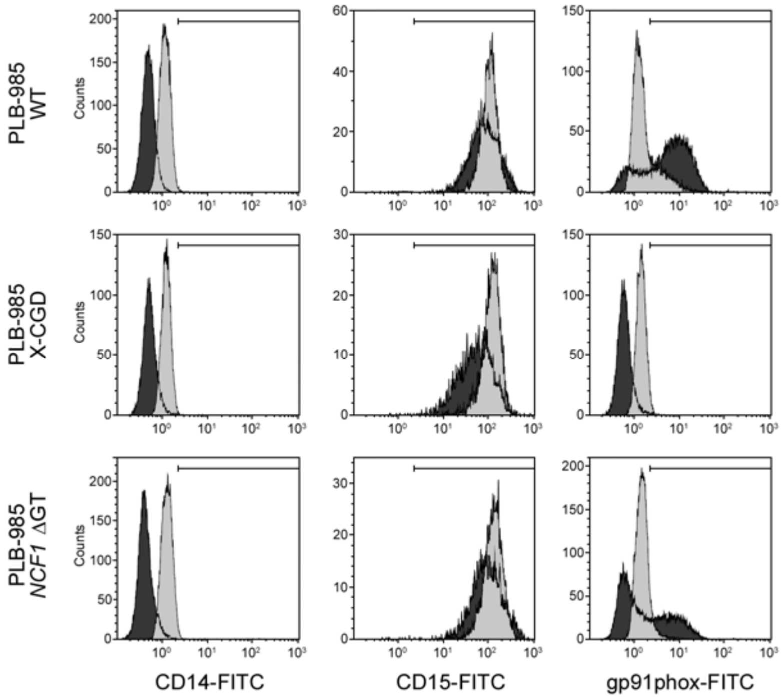

Flow cytometry analysis of PLB-985 WT, PLB-985 X-CGD and of PLB-985 *NCF1* ΔGT cells for CD14, CD15 and gp91phox expression.

All cell lines, PLB-985 WT, PLB-985 X-CGD, and PLB-985 *NCF1* ΔGT, were CD14 negative, CD15 positive in the surface marker analysis of CD11b positive (dark), and CD11b negative (bright). Upon differentiation 72 % of PLB-985 WT and 35 % of PLB-985 *NCF1* ΔGT cells displayed gp91^{phox} cell surface presentation.

Supplementary Figure S5

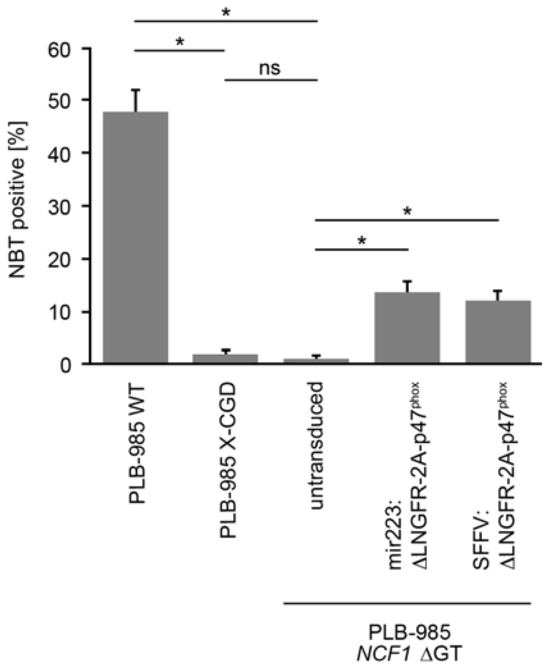

Retroviral reconstitution of ROS production in PLB-985 *NCF1* ΔGT cells.

Percentages (+/-SD) of ROS producing cells were determined by NBT assay in untransduced WT PLB-985 and PLB-985 X-CGD as well as in transduced and untransduced PLB-985 *NCF1* ΔGT cells upon differentiation to granulocytes (Figure 2B and E). Statistical analysis by one-way ANOVA with Tukey post hoc test was performed using SPSS software version 23. Ns: $P > .05$, *: $P < .001$.

Supplementary Figure S6

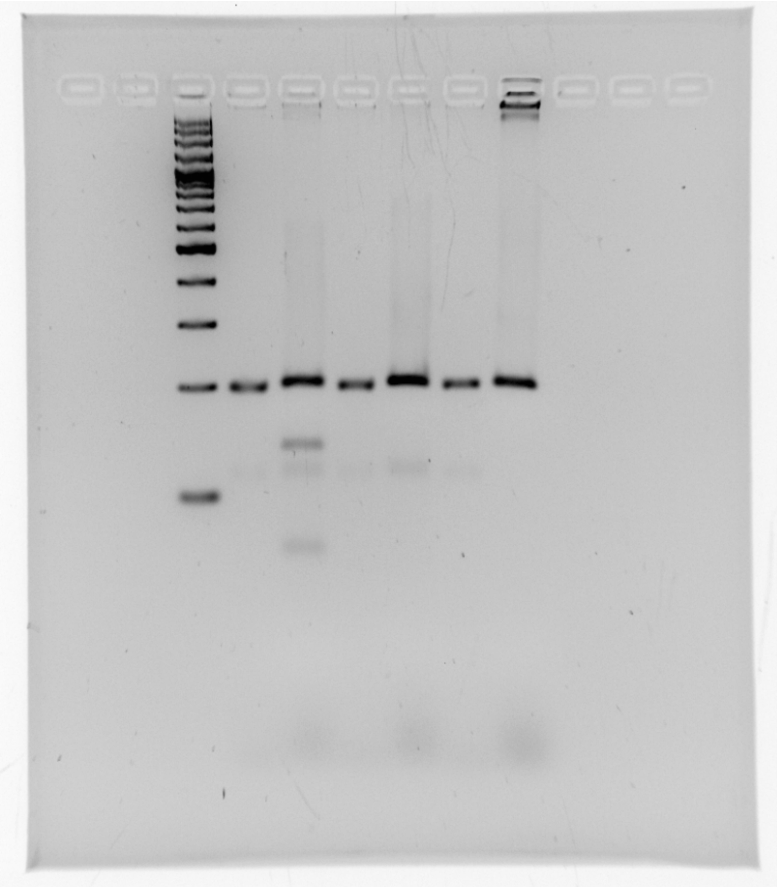

Loss of BsrG1 restriction site in PLB-985 *NCF1* ΔGT cells

Loss of BsrG1 restriction site in PCR amplified *NCF1 (B, C)* exon 2 (primers Fwd1 and Rev1 (Fig. 1B)) in PLB-985 *NCF1* ΔGT cells. Uncropped gel corresponding to Fig. 1D.

Supplementary Tables

Supplementary Table S1

**Generation of PLB-985 *NCF1* ΔGT cell line**		
PLB-985 WT cell line	Cell number	%
Nucleofected with pPX458-NCF1	$4.00 \cdot 10^6$	100.0
Nucleofection survival	$0.48 \cdot 10^6$	11.8 ± 1.7*
GFP expressing cells	$0.09 \cdot 10^6$	2.3 ± 1.0*
Sorted monoclonal cell lines	609	1.5 · 10-2
Sorting survival	22	5.5 · 10-4
Complete loss of BsrG1 restriction site	1	2.5 · 10-5

* Mean of two experiments with standard deviation of the mean.

Supplementary Table S2

Predicted off-target sites analyzed by Surveyor assay								
#	off-target sequence	score	mismatches (MMs)	UCSC gene	locus	primer forward	primer reverse	PCR product size (bp)
1	GCGCAAGATGTACATGTTCCTGG	1.4	4MMs [1:3:5:8]	NM_001190482	chr9:-78907149	TCTGAATCTTGGTAACCCATGG	GAGAGAAATAGGCTGAGATGATGAC	968
2	AGCACAGGTTTACATGTTCCTAG	1.3	4MMs [1:2:4:10]		chr17:+66815215	AGGCTGGAAGGAGGTTTTACT	AGTGAGTAGCGAATGGCCAGTAA	938
3	ATCCCACATGTACATGTTCCCAG	0.9	4MMs [1:2:7:8]		chr16:+65715259	AGTGGTGCAGGCCTATAGTC	AGATAATCAAAGCTGGCAGCTA	1049
4	AAACCATGTGTACATGTTCCCAG	0.9	4MMs [1:2:3:7]		chr1:-218029946	AATGCAGCCCAGGTCCTTTT	GATCCCTTGACCTCGTGTTCT	1407
5	CCTGCACCTGTACATGTTCCCAG	0.9	4MMs [3:4:7:8]		chr1:+8922827	GGGTAATATGGGAGAACTTAGC	GTTCTCCTCTGTGCTCTTGAC	1149
6	TCCTCAGTTGCACATGTTCCCAG	0.8	4MMs [1:4:8:11]		chr21:+35670707	GTGTGTTAAGTATTGGGTACTG	TGGCTAGAAATAATGTCTGGAA	957
7	CCACAAGGACTACATGTTCCTGG	0.8	4MMs [3:5:9:10]	NM_178003	chr9:+131899924	AGAGAGGCCTGCTCTGGTGA	GCAGTCACCAAGGCCAGTTAGT	915
8	GCTCCAGGTTCACATGTTCCCAG	0.7	4MMs [1:3:10:11]		chr1:+218024691	GAGAAGGATTTCCTGGAGGAAG	CCTCCTGAAAAGGCAAATACTC	986
9	CTCTCAGGTCAACATGTTCCAAG	0.7	4MMs [2:4:10:11]		chr3:-68375635	GTCACATAACATCTCTCAGCC	CTTCATAAGCTTATTCAGTTCCC	1074
10	CCCTCAAGTGTAGATGTTCCTAG	0.7	3MMs [4:7:13]		chr14:+22797496	GTGTTTAGTCTGAGACCCACC	GAGGTTATCTCCCAGGAACT	1141
11	CCTCCAGGCTGACATGTTCCTAG	0.4	4MMs [3:9:10:11]	NM_153613	chr15:-34653531	CAGCAGCTAAACCTGTACTG	GAATCTAGATACCTGGTCACTCT	903
12	CCCAGAGCTGTACAAGTTCCCGG	0.4	4MMs [4:5:8:15]	NM_031912	chr10:-46967666	CACTGTGTCCTTGAAACAGGT	CACGCTGAGGCCTGTCTTTT	995
13	CGCCCAGCTGTACATGATACAGG	0.2	4MMs [2:8:17:19]	NM_020845	chr12:-123519054	CCTGATCAGAGGCCACAATT	AGTGATAGATACCCTACAAGCACC	912
14	TCCCCAGTTGTGCATGTACCCGG	0.2	4MMs [1:8:12:18]	NM_004567	chr3:-48556455	ACAATGTGAAAGGAAAGTGCT	ATTACAAGTCCAGACACTTTCTGA	953
